# Identification and Biotechnical Potential of a Gcn5-Related N-Acetyltransferase Gene in Enhancing Microalgal Biomass and Starch Production

**DOI:** 10.3389/fpls.2020.544827

**Published:** 2020-08-28

**Authors:** Zhongze Li, Li Cao, Liang Zhao, Lihua Yu, Yi Chen, Kang-sup Yoon, Qiang Hu, Danxiang Han

**Affiliations:** ^1^Center for Microalgal Biotechnology and Biofuels, Institute of Hydrobiology, Chinese Academy of Sciences, Wuhan, China; ^2^College of Life Sciences, University of Chinese Academy of Sciences, Beijing, China; ^3^Laboratory for Marine Biology and Biotechnology, Qingdao National Laboratory for Marine Science and Technology, Qingdao, China; ^4^State Key Laboratory of Freshwater Ecology and Biotechnology, Institute of Hydrobiology, Chinese Academy of Sciences, Wuhan, China; ^5^Key Laboratory for Algal Biology, Institute of Hydrobiology, Chinese Academy of Sciences, Wuhan, China; ^6^The Innovative Academy of Seed Design, Chinese Academy of Sciences, Beijing, China

**Keywords:** starch, biomass, carbon partitioning, Gcn5-related N-acetyltransferase gene, *Chlamydomonas reinhardtii*

## Abstract

Microalgae are promising feedstocks for starch production, which are precursors for bioenergy and chemicals manufacturing. Though starch biosynthesis has been intensively studied in the green alga *Chlamydomonas reinhardtii*, regulatory mechanisms governing starch metabolism in this model species have remained largely unknown to date. We proposed that altering triacylglycerol (TAG) biosynthesis may trigger intrinsic regulatory pathways governing starch metabolism. In accordance with the hypothesis, it was observed in this study that overexpression of the plastidial lysophosphatidic acid acyltransferase gene (i.e. *LPAAT1*) in *C. reinhardtii* significantly enhanced TAG biosynthesis under nitrogen (N)-replete conditions, whereas the starch biosynthesis was enhanced in turn under N depletion. By the exploitation of transcriptomics analysis, a putative regulatory gene coding Gcn5-related N-acetyltransferase (*GNAT19*) was identified, which was up-regulated by 11–12 times in the *CrLPAAT1* OE lines. Overexpression of the cloned full-length *CrGNAT19* cDNA led to significant increase in the starch content of *C. reinhardtii* cells grown under both N-replete and N-depleted conditions, which was up to 4 times and 26.7% higher than that of the empty vector control, respectively. Moreover, the biomass yield of the *CrGNAT19* OE lines reached 1.5 g L^-1^ after 2 days under N-depleted conditions, 72% higher than that of the empty vector control (0.87 g L^-1^). Overall, the yield of starch increased by 118.5% in *CrGNAT19* OE lines compared to that of the control. This study revealed the great biotechnical potentials of an unprecedented *GNAT19* gene in enhancing microalgal starch and biomass production.

## Introduction

Many unicellular green algae can store carbon in the form of starch under adverse environmental conditions, such as high light and nutrient starvation (Ball et al., 1990). Microalgae are considered as promising feedstocks for producing starch, which can be used for bioethnol and chemicals manufacturing (Brányiková et al., 2011; Pancha et al., 2019a). However, regulatory mechanisms governing starch metabolism in microalgae have remained largely unknown to date, which is impeding development of rational genetic engineering strategy for enhancing starch production in these highly efficient photosynthetic cell factories.

Biochemical process of starch biosynthesis is conserved among most green algae and plants (Ball and Morell, 2003). The ADP-glucose pyrophosphorylase (AGPase, EC 2.7.7.23) is the first committed enzyme that catalyzes the formation of ADP-glucose, the nucleotide sugar donor for starch biosynthesis, from Glc-1-P and ATP in the chloroplasts. The formed ADP-glucose is then utilized by starch synthase (SS, EC: 2.4.1.21) as the building block to form the linear sugar polymers with more or less branches. Two types of SS have been identified, which are designated as soluble SS and granule-bound SS (GBSS). The soluble SS is generally believed to be responsible for synthesis of amylopectin, a type of highly branched starch molecules that constitute the major component of starch granules in green algae and plants. The GBSS is involved in biosynthesis of amylose, which is less branched starch molecules and is the minor component of starch granules. Synthesis of α-(1,6) branches in amylopectin is mediated by the starch branching enzyme (SBE, EC: 2.4.1.18), which hydrolyzes the α-(1,4) linkage and then catalyzes the formation of α-(1,6) linkage between the reducing end of the cut glucan and another glucosyl moiety of ADP-glucose. Debranching enzymes (DBE), including isoamylase (ISA, EC: 3.2.1.68) and limit-dextrinase (LDA, EC: 3.2.1.142), are involved in cleaving the branch points of amylopectin molecules, which play important role in shaping the structure of starch molecules.

In microalgae, degradation of starch not only occurs in dark to provide sugars for sustaining cellular metabolism and growth, but also plays an essential role in starch turnover under adverse environmental conditions, which can provide carbon skeleton for lipid biosynthesis (Roessler, 1988). Degradation of starch is initiated by the hydrolysis of sugar polymers on the surface of starch granules. The only enzyme has the capability to catalyze the reaction in such a solid region is the α-amylase (AMY, EC: 3.2.1.1), though the direct evidence for its involvement in degradation of starch granules in the plastids are still lacking. The linear chain of starch is primarily hydrolyzed by β-amylase (BAM, EC: 3.2.1.2). However, BAM cannot hydrolyze the branch point and the linkage immediately adjacent to it. Thus, complete breakdown of amylopectins requires the action of DBE, which can release short, linear, malto-oligo-saccharides into the chloroplast stroma. Degradation of starch also requires reversible phosphorylation of the starch molecules, which are catalyzed by glucan, water dikinases (GWD, EC 2.7.94) and phosphoglucan, water dikinases (PWD, EC 2.7.9.5).

The unicellular green alga *Chlamydomonas reinhardtii* is considered as an ideal model system to study the starch metabolism, due to the easiness of starch induction by simple high-light exposure or nutrient starvation. Iodine staining-assisted high throughput mutant screening enabled identification of a large number of gene loci involved in starch biosynthesis, including those coding for the AGPase (i.e. *SAT1* and *STA6*), GBSS1 (i.e *STA2*), SSS II (i.e. *STA3*), isoamylase (i.e. *STA7*) and PGM (i.e. *STA5*) and α-1,4 glucanotransferase (i.e. *STA11*) (Buléon et al., 1998; Wattebled et al., 2003). In addition, genome-wide survey revealed like plants *C. reinhardtii* also possesses multiple isoforms of many key enzymes involved in starch biosynthesis and turnover (Koo et al., 2017). Despite the fact that starch biosynthesis has been intensively studied in *C. reinhardtii*, the regulation of starch metabolism in this organism are still as poorly understood as that in most photosynthetic organisms.

Previous studies have indicated that starch biosynthesis interact with triacylglycerol (TAG) biosynthesis by sharing common carbon precursors in microalgal cells (Wang et al., 2009; Li et al., 2010a). In addition, it was found that blocking starch biosynthesis upregulated the expression of a number of genes involved in central carbon metabolism, leading to the increased carbon flow toward hexose-phosphate pathway (Blaby et al., 2013). Based on these findings, we hypothesized that altering TAG biosynthesis may trigger intrinsic regulatory pathways governing starch metabolism, and vice versa. By exploitation of a strategy combining TAG biosynthesis manipulation and transcriptomics analysis, given key genes involved in starch biosynthesis regulation could be identified.

In this study, it was observed that the transgenic *C. reinharditii* strains with overexpression of the plastidial lysophosphatidic acid acyltransferase (LPAAT1) exhibited the phenotype in accordance with our hypothesis. LPAAT catalyzes the acylation of lysophosphatidic acid (LPA) to produce phosphatidic acid (PA), which is the second step in the Kennedy pathway responsible for glycerolipids biosynthesis (Ohlrogge and Browse, 1995). CrLPAAT1 has been functionally characterized and was found to be localized in the chloroplasts of *C. reinhardtii* (Yamaoka et al., 2016). Overexpression of CrLPAAT1 significantly enhanced the TAG contents under N-replete conditions. When the OE lines were subject to N-depleted conditions, the starch biosynthesis was dramatically triggered instead, and meanwhile the TAG biosynthesis was relatively slowed down. By the exploitation of transcriptomics analysis and *in vivo* functional characterization, a Gcn5-related N-acetyltransferase (*GNAT*) gene was found to be involved in regulation of starch biosynthesis in *C. reinhardtii*. Additionally, overexpression of this gene can drastically enhance the microalgal biomass, indicative of its great potential in genetic engineering of microalgae for production of starch and other bioproducts.

## Materials and Methods

### Strains and Culture Conditions

The *C. reinhardtii* strains CC-400 and CC-124 were obtained from the *Chlamydomonas* Resource Center (http://chlamycollection.org/, Minnesota University). These reference strain was maintained respectively in 250 ml Erlenmeyer flask containing 100 ml tris-acetate-phosphate (TAP) liquid culture medium (Gorman and Levine, 1965) under continuous light (40 μmol photons m^-2^s^-1^) at 24 °C in the Innova incubators (New Brunswick Scientific) with agitation at 180 rpm.

The *CrLPAAT1* overexpression (OE) lines and empty vector (EV) controls were grown in 1,000 ml glass columns containing 800 ml Sueoka’s high salt (HS) medium (Sueoka, 1960) at an initial concentration of 2 × 10^5^ cells ml^-1^. The algal cells from the exponential phase (day 4, 2 × 10^7^ cells ml^-1^) were collected by centrifugation at 3,000 *g* for 5 min at room temperature and then transferred to the HS growth medium without the addition of ammonium chloride (referred to as N-free HS) after being washed with the growth medium. For both N-replete and N-depleted conditions, the algal cells were grown under continuous illumination (40 μmol photons m^-2^s^-1^) at 24°C with aeration of 1.5% (*v/v*) CO_2_.

The *CrGNAT19* OE lines and EV controls were cultured in 250 mL Erlenmeyer flask containing 100 ml TAP medium at an initial concentration of 2 × 10^5^ cells ml^-1^. The algal cells from the exponential phase (day 3, 2 × 10^7^ cells ml^-1^) were collected by centrifugation at 3,000 *g* for 5 min at room temperature and were transferred to the TAP growth medium without the addition of ammonium chloride (referred to as N-free TAP) after being washed with the growth medium. For both N-replete and N-depleted conditions, the algal cells were grown under continuous illumination (40 μmol photons m^-2^s^-1^) at 24 °C in Innova incubators (New Brunswick Scientific) with agitation at 180 rpm.

### Biomass Determination

The growth of algal cells was monitored by both dry cellular weight (DCW) and cell concentration measurement. DCW was determined by filtering algal cell cultures (10 ml) with the pre-weighed (W_0_) Whatman GF/B filters (ϕ 4.7 cm), which was then washed with 1 M ammonium formate solution to remove excess salts and then dried at 105°C by overnight. The weights of the filter with algal cells were recorded as W_1_, and the DCW per milliliter was determined as (W_1_-W_0_)/10. The cell concentrations used for growth curve establishment were determined as the optical density at 680 nm, which was referred to as OD_680_.

### Gene Cloning and Transgenic Strains Construction

The full-length cDNA of *CrLPAAT1* and *CrGNAT19* was amplified from the homemade cDNA library by using the primer sets *CrLPAAT1*-F (5’-ATGGCGCGTAAAAGCAGTTTGGCTC-3’)/*CrLPAAT1*-R (5’-CTGCTCATCCGGCGCCATCTCTGT-3’) and *CrGNAT19*-F (5’-ATGTCGGTCGTCAAATTACGCAAC-3’)/*CrGNAT19*-R (5’-CTACGCCAACCGCTTGTGCAT-3’), respectively. The PCR reactions were carried out by using Phanta Master Mix high-fidelity DNA polymerase (Vazyme, China) according to the manufacture’s instructions. To integrate the *CrLPAAT1* and *CrGNAT19* cDNA into the pChlamy_4 vector (Invitrogen, USA), the inserts with EcoRI-XbaI restriction sites were generated by using the primer sets *CrLPAAT1*-EcoRI-F (5’- GGAATTCATGGCGCGTAAAAGCAGTTTGGCTC-3’)/*CrLPAAT1*-XbaI-R (5’-GCTCTGAACTGCTCATCCGGCGCCATCTCTGT-3’) and *CrGNAT19*-EcoRI-F (5’-GGAATTCATGTCGGTCGTCAAATTAACGCAAC-3’)/*CrGNAT19*-XbaI-R (5’-GCTCTAGAACTACGCCAACCGCTTGTGCAT-3’), respectively. After digestion of the pChlamy_4 empty vector and inserts with EcoRI (Thermo Scientific, USA) and XbaI (Thermo Scientific, USA), the plasmids pChlamy4-*CrLPAAT1* and pChlamy4-*CrGNAT19* were constructed by using T4 DNA ligase (Promega, USA).

The constructed plasmid pChlamy4-*CrLPAAT1* and empty vector control was transformed into the reference strain CC-400, respectively, by using the glass-beads method (Kindle, 1990). Briefly, the algal cells were harvested at the exponential phase (2 × 10^6^ cells ml^-1^) by centrifugation at 1,500 g for 5 min. After discarding the supernatants, the cell pellets were resuspended in the pre-chilled TAP medium, while adjusting the final concentration to 2×10^8^ cells ml^-1^. Three-hundred microliters of cell suspension mixed with 300 mg sterile glass beads and 2 μg linearized plasmid DNA were vortexed for 20 s at the top speed. The transformed algal cells were recovered in 5 ml TAP medium under low light with continuous shaking (60–80 rpm) for 12 h. After concentration, the algal cells were resuspended in 200 μl TAP medium and spread onto the fresh agar plates containing TAP growth medium with zeocin (50 mg L^-1^). After growing under continuous light (40 μmol photons m^-2^ sec^-1^) at 25°C for 5 days, the transformants appeared and were verified with PCR.

The constructed plasmid pChlamy4-*GNAT19* and empty vector control was transformed into the reference strain CC-124 by using the electroporation method (Shimogawara et al., 1998), respectively. Briefly, the algal cells were harvested at the mid-log-phase (2 × 10^6^ cells ml^-1^) by centrifugation at 1,500 g for 5 min. After discarding the supernatants, the cell pellets were resuspended in the pre-chilled TAP media containing 60 mM sorbitol, while adjusting the final concentration to 2×10^8^ cells ml^-1^. Each electroporation cuvette with 250 μl cell suspension was incubated on ice for 10 min, and then added with 1 μg linearized plasmid DNA. Electroporation was conducted by using Bio-Rad electroporation system (Bio-Rad Gene Pulser Xcell Electroporation System, USA) under the optimized conditions (i.e. 400 V, 800 Ω, 50 μF). After electroporation, the cuvettes were immediately placed on ice for 10 min. The transformed algal cells were recovered in 10 ml TAP media containing 60 mM sorbitol under low light with continuous shaking (60–80 rpm) for 12 h. After centrifugation, the algal cells were resuspended in 200 μl TAP medium containing 60 mM sorbitol and 0.4% (*w*/*v*) PEG 6000 and spread onto the fresh agar plates containing TAP growth medium with paromomycin (50 mg L^-1^). After growing under continuous light (40 μmol photons m^-2^ sec^-1^) at 25°C for 5 days, the transformants appeared and were selected for PCR confirmation.

### RNA Extraction, Quantitative Real-Time PCR (qRT-PCR)

Algal cells were harvested by centrifugation at 1,000 *g* for 5 min and washed with the PBS buffer. The cell pellets were freeze-dried and stored at -80°C prior to use. Total RNA was isolated from the samples with TransZol Up Plus RNA kit (TransGen, China) by following the manufacturer’s instructions. One microgram of the total RNA was used as the template for cDNA synthesis performed by using PrimeScript II 1st Strand cDNA Synthesis Kit (Takara, Japan) with oligo (dT) primers. The qRT-PCR was carried out with LightCycler 96 (Roche, Switzerland). Each reaction system (20 μl) contained 100 ng cDNA template, 10 μM forward primer and reverse primer 1 μl, respectively and 10 μl LightCycler 480 SYBR Green I Master (Roche, Switzerland). The primer sets for *CrLPAAT1* and *CrGNAT19* was *CrLPAAT1*-QF (5’- ACATCTACTCGCTGTTCCACCTG-3’)/*CrLPAAT1*-QR (5’- CGGTCCACACGGTTTATCATCAC-3’) and *CrGNAT19*-QF (5’- AACGAGTACGGCTCCATCA-3’)/*CrGNAT19*-QR (5’- CTTCTGCTTCAACGCATCCA-3’), respectively. The *α-Tubulin* gene and *RACK1* gene were used as internal references, which was amplified by using the primer set *Tubulin*-F (5’-CTCGCTTCGCTTTGACGGTG-3’)/*Tubulin*-R (5’- CGTGGTACGCCTTCTCGGC-3’) and *RACK1*-F (5’- TTCTCGCCCATGACCACCAAC-3’)/*RACK1*-R (5’- GGCCCACCAGGTTGTTCTTCAG-3’), respectively. The amplification conditions were as follows: 95°C for 10 min, followed by 40 cycles of 15 sec at 95°C, 30 s at 60°C and 30 s at 72°C. The specificity of qRT-PCR amplification was checked with the melting curve program (95°C for 10 s, 65°C for 60 s, continuous acquisition at 97°C for 1 s and cooling at 37°C for 30 s). The normalization and relative expression of *CrLPAAT1* and *CrGNAT19* genes were determined by geometric averaging of two internal reference genes as 2^-ΔΔCt^ (Livak and Schmittgen, 2001; Vandesompele et al., 2002).

### Western Blotting Analysis

Three milliliters of algal cells were harvested by centrifugation at 1,000 *g* for 5 min, cell pellets were resuspended in 120 µl Buffer A (0.1 M dithiothreitol, 0.1 M Na_2_CO_3_) and 80 µl Buffer B (5% SDS, 30% sucrose, *w*/*v*). Cell suspension was vortexed for 30 min and lysed two cycles of freeze/thawing. After that, lysates were centrifuged at 16,000 *g* for 10 min, the supernatant was transferred to a new tube. The total protein concentration was measured by using CB-X protein assay kit (G-Biosciences, USA).

Around 20 µg of proteins in the SDS-PAGE sample buffer were separated on 12% (*w*/*v*) SDS-PAGE gel, and transferred to polyvinylidene fluoride (PVDF) membrane at 1.3 A, 25 V, for 7 min by using semi-dry transfer system (Bio-Rad Trans-Blot Turbo Transfer System, USA). The membranes were blocked with 10 ml of 5% nonfat milk. For immunofluorescence detection of CrGNAT19 and CrLPAAT1, the membrane was incubated overnight with 6×His-tag antibody conjugated with horseradish peroxidase (Proteintech, China, 1:10,000 dilution) and rabbit primary anti-CrLPAAT1 (1:2,000 dilution) with TBS containing 2% (*w/v*) nonfat milk, respectively, followed by incubation with the secondary antibody anti-rabbit IgG conjugated with horseradish peroxidase diluted by 1:1,000. Antigen-antibody complexes were visualized using an enhanced chemiluminescence detection kit (Bio-Rad Clarity Western ECL Substrate, USA). The Tubulin was used as a reference, which was detected by using the Tubulin alpha chain antibody (Agrisera, Sweden, 1:1,000 dilution).

The CrLPAAT1 antibody was generated by using the synthetic peptide. Briefly, a peptide (VHKALPPNKNADQL) was selected based on the screening of the antigenic epitopes of CrLPAAT1 (Protean program, LaserGene software). The peptide was synthesized and then coupled with bovine serum albumin (BSA) (YouLong Biotech, China). After immunizing the rabbit for four times over three months, the recovered antiserum was used for purification of the CrLPAAT1 polyclonal antibody by affinity chromatography (YouLong Biotech, China).

### RNA-Seq

Five milliliters of the *CrLPAAT1* OE and EV control cell cultures grown under N-replete and N-depleted conditions were collected on 0, 3, and 12 h for RNA isolation. For each time point, 4 biological replicates were prepared. Total RNA were extracted by using Trizol reagents (Invitrogen, USA), from which the poly(A)-containing mRNA molecules were purified by using NEBNext Poly(A) mRNA Magnetic Isolation Module (New England Biolabs, USA). Directional transcriptome libraries were prepared by using NEBNext Ultra Directional RNA Library Prep Kit for Illumina (New England Biolabs, USA) which adopts the “dUTP” method to generate the strand-specificity library (Parkhomchuk et al., 2009). The constructed libraries were sequenced for 2×150-bp runs (paired-end) by using HiSeq 3000 (Illumina, USA). To ensure quality, adapter pollutions and low-quality reads were deleted from the raw data with Trimmomatic (version 0.35) (Bolger et al., 2014). For each data set, gene expression was measured as the numbers of aligned reads to the transcript assembly by using the alignment-based quantification method RSEM (Mortazavi et al., 2008), which provided the counts of RNA-Seq fragments and normalized expression metrics as “transcripts per million transcripts” (TPM). A given gene was considered to be significantly differentially expressed if the following criteria were met: i) there was at least a 2-fold change with a false discovery rate (FDR)-corrected P value ≤0.05 between control and stress conditions; ii) the counts-per-million (CPM) values under both conditions were ≥5. for, their values under each condition were ≥5.

### Phylogenetic Analysis

The amino acid sequences of the selected proteins were aligned by using the Clustal W program built in MEGA 6.0 (Thompson et al., 1994). The phylogenetic tree was generated by using the neighbor-joining algorithm with MEGA 6.0. Molecular distances within the aligned sequences were calculated according to the position correction model. The confidence of each clade was tested through bootstrapping analysis with 1,000 replications.

### Biochemical Composition Analysis

The cellular contents of the total fatty acids were quantified according to the methods described previously (Wu et al., 2019). To measure the TAG contents, total lipids were extracted from 10 mg of the lyophilized algal biomass. Briefly, the cell pellets were mixed with 4 ml of MeOH/CHCL_3_/80% (*v*/*v*) HCOOH (20:10:1, *v*/*v*/*v*). After vortex for 1 h, 2 ml of salt solution (1M KCL, 0.2M H_3_PO_4_) was added to the mixture to partition the organic and aqueous phase. The organic phase was further transferred to a new vial and was dried under N_2_ stream. TAG was separated *via* thin layer chromatography (TLC). The TAG spots were scraped from the TLC silico gel glass plate and then trans-esterified to the fatty acid methyl esters for gas chromatography mass spectrometry analysis (Wu et al., 2019). Ten milligrams of lyophilized algal biomass of each cell lines were used to determine the total carbohydrate content by using the phenol-sulfuric acid method described previously (Dubois et al., 1956; Jia et al., 2015). The starch contents were quantified by using the total starch assay kit (Megazyme International, Ireland) according to the manufacturer’s directions. The total protein contents were measured by using the Bradford protein assay method (Berges et al., 1993; Jia et al., 2015).

### Statistical Analysis

A student’s t-test was used to prove statistical difference (*, *p* < 0.05; **, *p* < 0.01; ***, *p* < 0.001).

## Results

**Figure 1:**
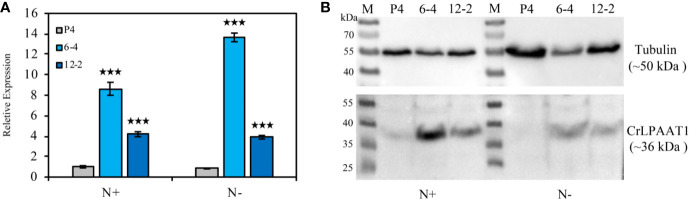
Construction of the *CrLPAAT1* OE lines (6-4 and 12-2) and empty vector control (P4). **(A)** Relative transcript levels of the *CrLPAAT1* encoding genes in the empty vector line (P4) and two *CrLPAAT1* OE lines (normalized to that of P4) under nitrogen replete and nitrogen depletion conditions. **(B)** Protein levels of the empty vector line (P4) and two *CrLPAAT1* OE lines under nitrogen replete and nitrogen depletion conditions. Around 20 μg of whole-cell protein were loaded in each lane. Values are the means ± S.D. (n = 4). Student’s t-test (***p < 0.001).

**Figure 2:**
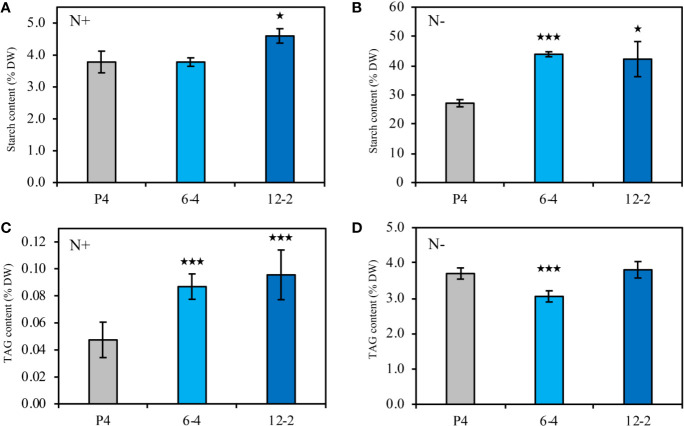
Biochemical compositions of the *CrLPAAT1* OE lines (6-4 and 12-2) and empty vector control (P4). **(A)** Starch contents (% DW) in P4 and two *CrLPAAT1* OE lines (6-4, 12-2) under nitrogen replete condition. **(B)** Starch contents (% DW) in P4 and two *CrLPAAT1* OE lines (6-4, 12-2) under nitrogen depletion condition. **(C)** TAG contents (% DW) in P4 and two *CrLPAAT1* OE lines (6-4, 12-2) under nitrogen replete condition. **(D)** TAG contents (% DW) in P4 and two *CrLPAAT1* OE lines (6-4, 12-2) under nitrogen depletion condition. Values are the means ± S.D. (n = 4). Student’s t-test (*p < 0.05; ***p < 0.001).

In this study, two *CrLPAAT1* OE lines (i.e. 6-4 and 12-2) were obtained, in which the transcripts and protein levels of *CrLPAAT1* dramatically increased as compared to that of the empty vector P4 (**Figure 1**). It is noteworthy that both the transcript and protein level of the P4 empty vector line decreased under N-depletion condition as compared to that under N-replete conditions. When the algal cells were grown under the normal N-replete conditions, 2-fold increases in the TAG contents were observed in both the *CrLPAAT1* OE lines (**Figure 2C**). After being subject to N-depletion stress for 3 days, however, the TAG content of the OE line 6-4 was 10% lower (*p* < 0.001) than that of the control, and no significant difference was observed between the OE line 12-2 and P4 (**Figure 2D**). According to the hypothesis, the enhanced TAG biosynthesis in *CrLPAAT1* OE lines grown under N-replete conditions may in turn stimulate starch biosynthesis. Accordingly, the starch contents were found elevated by 50% in the two OE lines under N-depleted conditions (**Figure 2B**), though it slightly increased in one of the OE lines under N-replete conditions (**Figure 2A**).

To unravel the yet unknow regulatory mechanism governing carbon partitioning, the transcriptomic analysis was performed on the *CrLPAAT1* OE lines and EV control. Thirty-six high-quality transcript profiles were generated, featured by high reproducibility (Spearman correlation > 0.95) among the four biological replicates at each time point. Under the N-replete condition (0 h), there were 4,055 up-regulated and 3,256 down-regulated genes in the *CrLPAAT1* OE lines. At the time point of 3 h after subject to N depletion, 3,818 and 3,165 genes were up- and down-regulated, respectively. After 12 h under N depletion condition, there were 3,547 up-regulated and 3,358 down-regulated genes in the *CrLPAAT1* OE lines. Thus, it can be concluded that overexpression of *CrLPAAT1* led to profound changes in various biological processes at transcript level.

**Figure 3:**
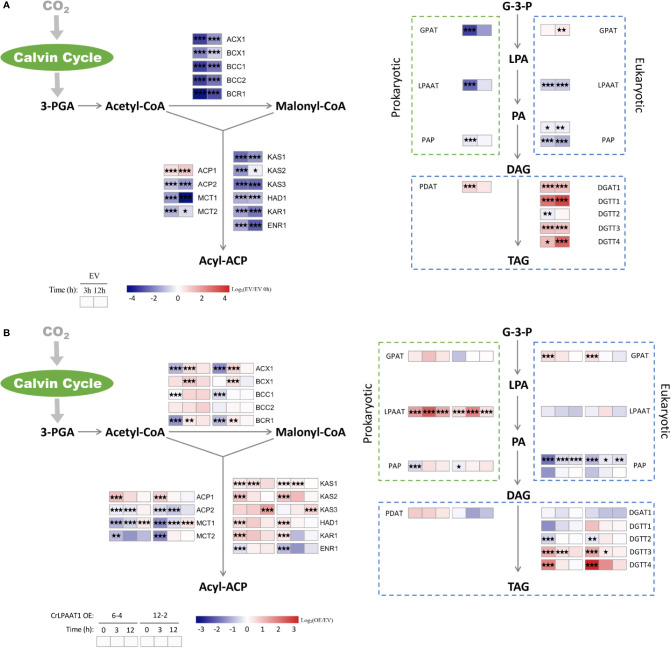
Alterations in the gene expression levels in the fatty acids *de novo* biosynthesis and TAG assembly pathways of the P4 empty vector lines and *CrLPAAT1* OE lines (6-4 and 12-2). **(A)** Fold-changes of P4 empty vector lines over 12 h upon the onset of N-depletion stress were calculated as log_2_ (EV/EV 0 h) (EV, P4 empty vector lines). **(B)** Fold-changes between *CrLPAAT1* overexpression lines and P4 empty vector lines over 12 h upon the onset of N-depletion stress were calculated as log_2_ (OE/EV) (OE, *CrLPAAT1* overexpression lines; EV, P4 empty vector lines) and displayed in the heat map. 3-PGA, 3-phosphoglyceric acid; *ACX*, α-carboxyltransferase (ACCase complex); *BCX*, β- carboxyltransferase (ACCase complex); *BCC*, acetyl-CoA biotin carboxyl carrier protein (ACCase complex); *BCR*, biotin carboxylase (ACCase complex); *ACP*, acyl carrier protein; *MCT*, malonyl-CoA: ACP transacylase; *KAS*, 3-ketoacyl-CoA-synthase (FAS complex); *HAD*, 3-hydroxyacyl-ACP dehydratase (FAS complex); *KAR*, 3-oxoacy-[acyl carrier protein] reductase (FAS complex); *ENR*, enoyl-[acyl carrier protein] reductase (FAS complex); G-3-P, glycerol-3-phosphate; LPA, lysophosphatidic acid; PA, phosphatidic acid; DAG, diacylglycerol; *GPAT*, glycerol-3-phosphate acyltransferase; *LPAAT*, lysophosphatidic acid acyltransferase; *PAP*, phosphatidic acid phosphatase; *PDAT*, phospholipid: diacylglycerol acyltransferase; *DGAT*, diacylglycerol acyltransferase type 1; *DGTT*, diacylglycerol acyltransferase type 2. Statistical significances of the differentially expressed genes in the P4 empty vector lines and the *CrLPAAT1* overexpression lines with same trend of regulation between two *CrLAAT1* OE lines were determined by a false discovery rate (FDR)-corrected p value (*p < 0.05; **p < 0.01; ***p < 0.001).

The fold-changes of the transcripts involved in fatty acid and lipid biosynthesis were shown in **Figure 3**. In the EV control, most genes involved in fatty acid biosynthesis were significantly down-regulated at the transcript level in response to N depletion, except for one of the acyl-carrier protein (ACP) encoding genes (**Figure 3A**). When comparing the *CrLPAAT1* OE lines with the EV control, it was found that five genes in the fatty acids *de novo* biosynthesis pathways exhibited downregulation at transcript level under N-replete conditions, including those coding biotin carboxylase (BCR), acyl-carrying protein (ACP2), malonyl-CoA:acyl protein malonyltransferase (MCT1 and MCT2), and enoyl-ACP reductase (ENR1) (**Figure 3B**). Among them, *ACP2* and *MCT2* were down-regulated over 3 h after being subject to N depletion, whereas the transcription of *BCR1*, *ACP2* and *MCT1* were conversely up-regulated under N-depleted conditions (**Figure 3A**, **Supplemental Table**). By contrast, three 3-ketoacyl-acyl carry protein synthase encoding genes (i.e. *KAS1*, *KAS2*, and *KAS3*) were significantly up-regulated in a sequential manner (**Figure 3A**, **Supplemental Table**). Besides, the up-regulated genes under N-depleted conditions included hydroxoacyl-ACP dehydrogenase (*HAD1*) and 3-ketoacyl-ACP (*KAR1*) (**Figure 3A**, **Supplemental Table**).

The synthesized fatty acids are assembled with the glycerol backbone to form TAG *via* two Kennedy pathways, which is so-called prokaryotic and eukaryotic pathway, respectively (Fan et al., 2011). Both the pathways are involved in sequential acylation of glycerol-3-phostate and one step of dephosphorylation, but differ in their subcellular localizations, the former of which resides in plastids and the latter is associated with the endoplasmic reticulum membranes. In the EV control, four diacylglycerol acyltransferase (DGAT) genes and one phospholipid:diacylglycerol acyltransferase (PDAT) gene were significantly up-regulated over 12 h in response to N depletion (**Figure 3A**). Overexpression of CrLPAAT1 led to significant up-regulation of the eukaryotic glycerol-3-phosphate acyltransferase (*GPAT*) and two type II diacylglycerol acyltransferase encoding genes (*DGTT3* and *DGTT4*) under N-replete conditions (**Figure 3B**, **Supplemental Table**). However, none of the genes directly involved in TAG biosynthesis were induced at the transcript level in *CrLPAAT1* OE lines under N-depleted condition (**Figure 3A**, **Supplemental Table**).

**Figure 4:**
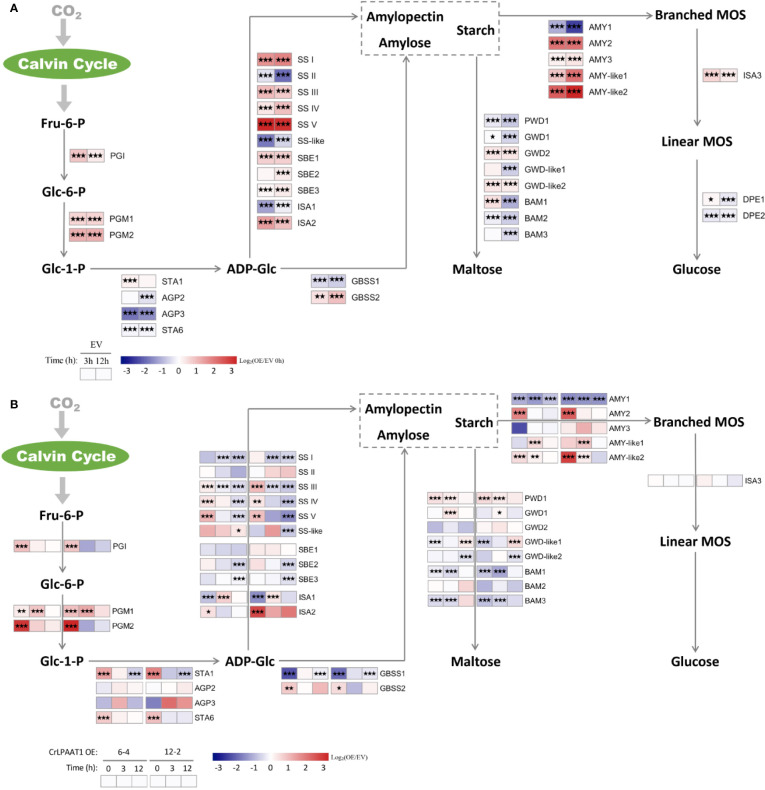
Alterations in the gene expression levels in the starch metabolism pathways of the P4 empty vector lines and *CrLPAAT1* OE lines (6-4 and 12-2). **(A)** Fold-changes of P4 empty vector lines over 12 h upon the onset of N-depletion stress were calculated as log_2_ (EV/EV 0 h) (EV, P4 empty vector lines). **(B)** Fold-changes between *CrLPAAT1* overexpression lines and P4 empty vector lines over 12 h upon the onset of N-depletion stress were calculated as log_2_ (OE/EV) (OE, *CrLPAAT1* overexpression lines; EV, P4 empty vector lines) and displayed in the heat map. Fru-6-P, fructofuranose 6-phosphate; Glc-6-P, glucopyranose 6-phosphate; Glc-1-P, glucopyranose 1-phosphate; ADP-Glc, ADP-glucose; Branched-MOS, branched malto-oligosaccharides; Linear-MOS, linear malto-oligosaccharides; *PGI*, phosphoglucose isomerase; *PGM*, phosphoglucomutase; *AGP*, ADP-glucose pyrophosphorylase; *SS*, starch synthase; *GBSS*, granule bound starch synthase; *SBE*, starch branching enzyme; *ISA*, isoamylase; *AMY*, α-amylase; *BAM*, β-amylase; *PWD*, phosphoglucan water dikinase; *GWD*, alpha-glucan water dikinase; *DPE*, Disproportionating enzyme. Statistical significances of the differentially expressed genes in the P4 empty vector lines and the *CrLPAAT1* overexpression lines with same trend of regulation between two *CrLAAT1* OE lines were determined by a false discovery rate (FDR)-corrected p value (*p < 0.05; **p < 0.01; ***p < 0.001).

The fold-changes in the transcripts involved in starch biosynthesis and degradation were shown in **Figure 4**. In the empty vector, a number of genes in the starch biosynthesis pathway were up-regulated in response to N-depletion. In addition to the phosphoglucose isomerase (PGI) and phosphoglucomutase (PGM) encoding genes responsible for biosynthesis of glucose-1-phosphate (Glc-1-P), up-regulated genes in the empty vector subject to N-depletion included *SS I/II/IV/V*, *SBE1/2*, *ISA2*, and *GBSS2* (**Figure 4A**). However, the large subunits of AGPase encoding gene *STA6* was significantly down-regulated, and *STA1* that codes the large subunit was only slightly up-regulated on 3 h in the EV control after being subject to N-depletion (**Figure 4A**). Overexpression of CrLPAAT1 led to the up-regulation of *STA6* and *STA1* under N-replete conditions: *STA6* was up-regulated by 4.2 and 6.8 times, and *STA1* was up-regulated by 2.8 and 3.1 times in 6-4 and 12-2, respectively (**Figure 4B**, **Supplemental Table**). Though the expression of *SS I/III/IV/V*, *SBE1/2*, *ISA2* and *GBSS2* at the transcript level were elevated in *CrLPAAT1* OE lines, their transcripts level were lower than that of the control under N-depleted conditions (**Figure 4B**, **Supplemental Table**).

Among the genes involved in the degradation of starch to glucose, *AMY2* and two *AMY-like* genes were substantially up-regulated, along with slight up-regulation of *AMY3* and *ISA3*, in the empty vector control in response to N-depletion (**Figure 4A**). Based on the gene expression data, it can be perceived that the degradation of starch into maltose was most likely reduced, as most genes in this pathway were down-regulated except for several slightly induced genes (e.g. *GWD2*, *GWD-like2*, and *BAM1*). In *CrLPAAT1* OE lines, two of the β-amylase genes (i.e. *BAM1* and *BAM2*) and one of the α-amylase genes were suppressed at the transcript level under both N-replete and N-depleted conditions. *AMY2* was considerably up-regulated by 6.9 and 13.5 times in the two OE lines, respectively. Similarly, the *AMY-like2* gene was up-regulated under both N-replete and N-depleted conditions in the two OE lines as well. The differentially expressed *PWD1* and *GWD1* showed up-regulation at the transcript level, the former of which was elevated by 2.5 and 2.2 times under N-replete and N-depleted conditions, respectively, and the latter one was up-regulated by ca. 2.2 times on 3 h after subject to N-depletion. However, the transcription of two *GWD1-like* genes were changed in an opposite way to that of *GWD1*, which was down-regulated under N-replete and N-depleted condition, respectively.

**Figure 5:**
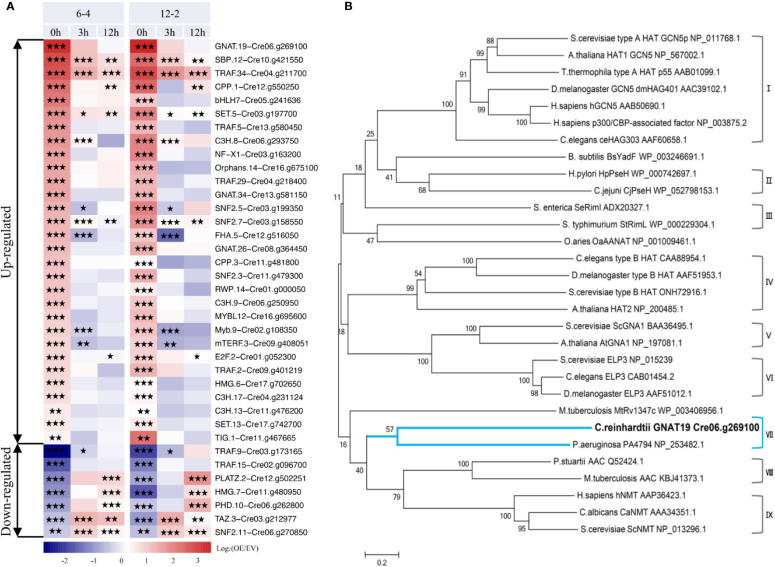
Identification of a putative non-repressible 5 (Gcn5)-related N-acetyltransferase (GNAT) encoding gene (i.e. Cre06.g269100A, referred to as *CrGNAT19* in this study). **(A)** Transcriptional dynamics of the differentially expressed transcription factor/regulator encoding genes in the two *CrLPAAT1* OE lines (i.e. 6-4 and 12-2). Fold-changes over 12 h upon the onset of N-depletion stress were calculated as log_2_ (OE/EV) (OE, *CrLPAAT1* overexpression lines; EV, P4 empty vector lines) and displayed in the heat map. The genes were classified into two groups including the up-regulated and down-regulated ones, according to the relative gene expression level at the time point 0 h. Statistical significances of the differentially expressed genes with same trend of regulation between two *CrLAAT1* OE lines were determined by a false discovery rate (FDR)-corrected p value (*p < 0.05; **p < 0.01; ***p < 0.001). **(B)** Phylogenetic tree of GNAT19 and related GNATs constructed by comparing amino acid sequences. The GNAT family consists of nine subgroups, I: Type A HATs, II: Pseudaminic acid biosynthesis protein H, III: Ribosomal protein Nα-acetyltransferases, IV: Type B HATs, V: Glucosamine-6-phosphate N-acetyltransferases, VI: Elongator complex protein, VII: C-terminal Nϵ-lysine protein acetyltransferases, VIII: Aminoglycoside N-acetyltransferases, IX: Protein N-myristoyltransferases. The tree was generated by using the neighbor-joining algorithm with MEGA 6.0. Molecular distances within the aligned sequences were calculated according to the position correction model. The confidence of each clade was tested through bootstrapping analysis with 1,000 replications.

Based on the analysis above, it can be concluded that overexpression of *CrLPAAT1* caused complicated transcriptional changes in the lipid and starch metabolism pathways, so that a higher-tier regulation could be expected, such as activation or deactivation of transcription factors (TFs) or regulators (TRs) in the *CrLPAAT1* OE lines. Moreover, enhanced starch biosynthesis in *CrLPAAT1* OE lines under N-depleted conditions could not be attributable to the transcriptional regulation. Thus, activation of genes involved in post-translational regulation could be another possible scenario. To verify such a hypothesis, the putative transcription factor/regulator genes were identified from the differentially expressed genes based on previous prediction (Gargouri et al., 2015). When compared with the EV control, a total of 37 putative TF/TR genes were differentially expressed in the two *CrLPAAT1* OE lines (i.e. 6-4 and 12-2). These genes fell into two clusters based on their fold-changes under N-replete conditions (**Figure 5A**). The up-regulated cluster included 30 genes, among which three genes were up-regulated under both conditions, whereas the other genes were unchanged or down-regulated under N-depleted conditions. The most enriched gene families in this cluster included *TRAF*, *C3H*, general control non-repressible 5 (Gcn5)-related N-acetyltransferase (*GNAT*), and *SNF*. The down-regulated cluster included 5 genes. It is noteworthy that most of the identified up-regulated TFs/TRs can be induced at the transcript level in the EV control (regarded as wild-type background) subject to N-depletion (**Figure S1**). On the top of the up-regulated genes, a putative general control non-repressible 5 (Gcn5)-related N-acetyltransferase (*GNAT*) gene (i.e. Cre06.g269100, named as *GNAT19* in this study) showed the most substantial up-regulation in the *CrLPAAT1* OE lines, which increased by 11 and 12 times in the two OE lines under N-replete conditions, respectively (**Figure 4A**). In addition, 5.3-fold increase in the expression of this gene was observed in the EV control on 12 h under N-depleted conditions (**Figure S1**).

To dissect the function of CrGNAT19, particularly for its potential in regulating carbon partitioning, the full-length cDNA of *CrGNAT19* was cloned from the cDNA library of *C. reinhardtii*. *In silico* analysis indicated that *CrGNAT19* codes for a protein consisting of 370 amino acid residues with a mass of 39.37 KD and a pI of 8.85. Phylogenetic analysis of *CrGNAT19* along with 29 *GNAT* with experimentally confirmed functions from various organisms revealed that CrGNAT19 was closely related with a C-terminal Nϵ-lysine protein acetyltransferases of the bacterium *Pseudomonas aeuruginosa* (Majorek et al., 2013) (**Figure 5B**).

**Figure 6:**
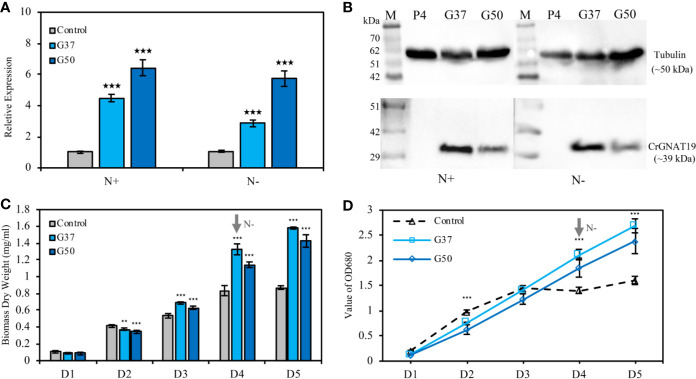
Characterization of the *CrGNAT19* OE lines. **(A)** Relative transcript levels of *CrGNAT19* in the two OE lines (i.e. G37, and G50) and empty vector control line (i.e. P4) under nitrogen replete and nitrogen depletion conditions. **(B)** Protein levels of *CrGNAT19* in the two OE lines (i.e. G37, and G50) and empty vector control line (i.e. P4) under nitrogen replete and nitrogen depletion conditions. **(C)** Dry weights of the two *CrGNAT19* OE lines and empty vector control (i.e. P4) under N-replete and N-depleted conditions for 5 days. **(D)** Cellular growth measured as the optical density absorbance at 680 nm under the N-replete and N-depleted conditions for 5 days. Values are the means ± S.D. (n = 4). Student’s t-test (**p < 0.01; ***p < 0.001).

Two *CrGNAT19* OE lines (i.e. G37 and G50) were constructed and verified with RT-PCR (**Figure 6A**) and western blotting (**Figure 6B**). Since *CrGNAT19* OE lines did not grow very well under the photoautotrophic conditions, of which the cells tended to aggregate and attach to the wall of photobioreactors, they were thus grown under the mixotrophic conditions for phenotyping analysis. Overexpression of CrGNAT19 slightly retarded the cellular division of *C. reinhardtii* during the exponential phase under N-replete conditions, whereas the biomass yield of *CrGNAT19* OE increased by 20% as compared to that of the EV control (**Figure 6C**). When the algal cells were subject to N-depletion, the *CrGNAT19* OE lines continued growing over 2 days, while the cell division of the control ceased upon exposure to the stress (**Figure 6D**). After 2 days under N-depletion, the biomass yield of the *CrGNAT19* OE lines reached 1.5 g L^-1^, which increased by 72.6% as compared to that of the control (**Figure 6C**).

**Figure 7:**
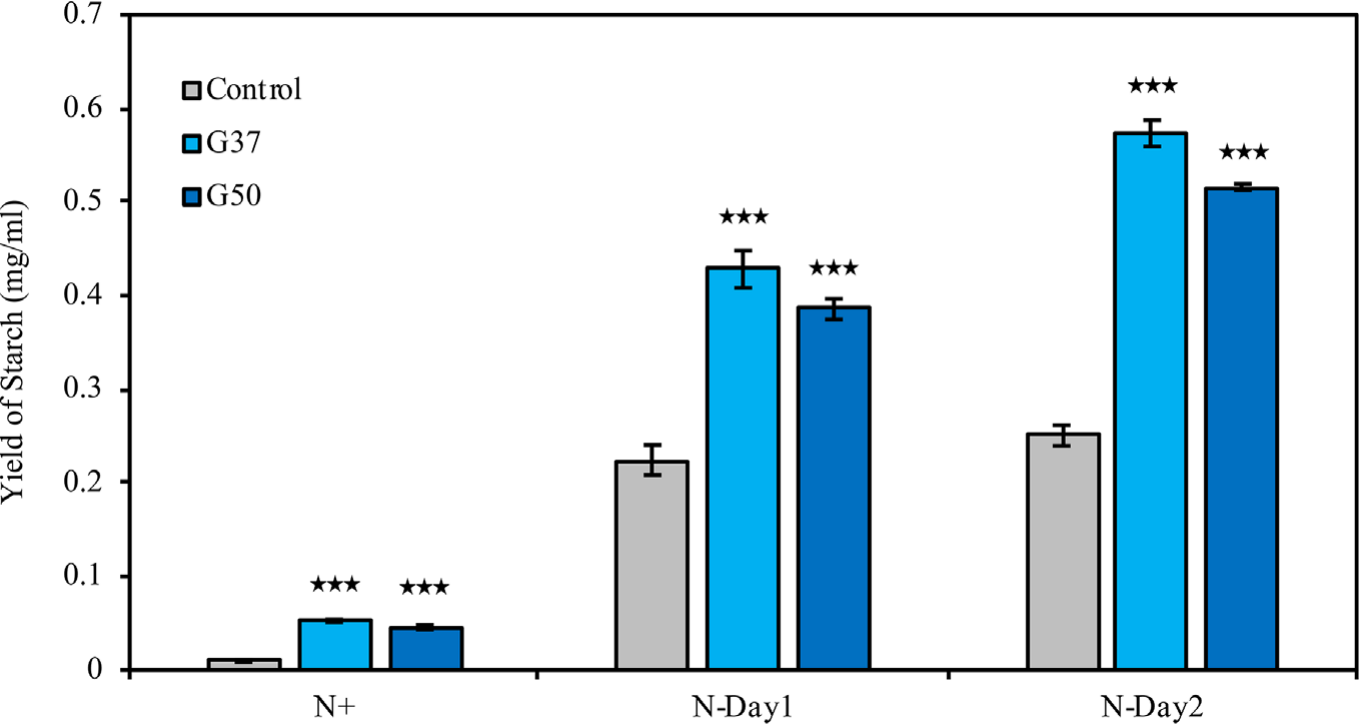
Enhanced starch yield in the *CrGNAT19* OE lines. Yield of starch in P4 control lines and two *CrGNAT19* OE lines subject to N- replete (N+) and N- depletion stress for two days (N-Day1, N-Day2). Values are the means ± S.D. (n = 4). Student’s t-test (***p < 0.001).

Biochemical composition analysis revealed that there were 2.7- and 1.3-fold increases in the total carbohydrate content of the *GNAT19* OE lines grown under N-replete and N-depleted conditions, respectively, when compared to that of the control (**Table 1**). As the major form of carbon storage in the algal cells, the starch content was enhanced from 1.8 to 7.5% of DCW in the *CrGNAT19* OE lines grown under N-replete conditions. Albeit both the *CrGNAT19* OE lines and control accumulated substantial amounts of starch in response to N-depletion, the starch content of the former reached 36.5% of DCW, which increased by 26.7% as compared to that of the latter (**Table 1**). In contrast to the enhanced starch biosynthesis, the content of total lipids and TAG was reduced by 34 and 67%, respectively, in the *CrGNAT19* OE lines subject to the N-depleted conditions. After 2 days under N-depleted conditions, the TAG content of the *CrGNAT19* OE lines was only 1.8% of DCW, significantly lower than that of the control (5% of DCW) (**Table 1**). Compared to the control, the final starch yield of the *CrGNAT19* OE lines, as a function of the starch content and biomass concentration, increased by 118.5% over 2 days under N-depleted conditions (**Figure 7**).

**Table 1:** Biochemical compositions of the *GNAT19* OE lines grown under the N-replete and N-depleted conditions for two days.

Content (% DW)	N-replete			N-depleted DAY1			N-depletedDAY2		
	Control	GNAT19	*p* value	Control	GNAT19	*p* value	Control	GNAT19	*p* value
Total fatty acids	5.76 ± 0.094	5.66 ± 0.060	0.082	8.18 ± 0.093	6.50 ± 0.16	<0.001	9.50 ± 0.31	6.25 ± 0.040	<0.001
TAG	0.044 ± 0.004	0.041 ± 0.005	0.330	2.68 ± 0.11	1.68 ± 0.13	<0.001	5.54 ± 0.59	1.79 ± 0.25	<0.001
Total carbohydrates	3.58 ± 0.45	9.72 ± 0.42	<0.001	33.64 ± 0.77	38.97 ± 1.50	<0.001	33.55 ± 0.72	44.04 ± 2.56	<0.001
Starch	1.83 ± 0.21	7.53 ± 0.23	<0.001	26.60 ± 0.36	33.83 ± 0.78	<0.001	28.83 ± 0.53	36.51 ± 1.44	<0.001

Values are the means ± S.D. (n = 4). Student’s t-test was used to determine the statistical significance indicated by the p value. DW, dry weight.

## Discussion

Many biotechnical efforts have been made to enhance the starch contents in a variety of crops and microalgae, mainly *via* increasing biosynthesis or decreasing breakdown of starches (Smith, 2008). One of the mostly reported targeted gene is that coding for AGPase, but its overexpression led to only marginal, in some cases, or even intangible impacts on the starch contents in many crops (Giroux et al., 1996; Meyer et al., 2007). The difficulties in identifying the appropriate gene targets for bioengineering are the complexity of the starch metabolism pathways. Given that a large number of isoforms are involved in starch biosynthesis, turnover, and degradation, their precisive roles in governing the accumulation of starches haven’t been fully understood yet, and on the other hand, manipulation of a single pathway may cause compensatory responses from its counterparts or alternative pathways. Such complexity was perceived in *C. reinhardtii* as well. In the *CrLPAAT1* OE lines, multiple isoforms of the most starch metabolism-related genes were differentially regulated at the transcriptional level when the starch accumulation was significantly enhanced (**Figure 4B**). Thus, the major aim of this study was to identify the regulatory gene(s) that can be leveraged for rational genetic engineering.

Regulation proteins such as TFs, TRs, as well as those involved in post-translational modification, are representing the most promising targets for rewiring carbon partitioning for enhancing production of starches or lipids in microalgae. Recently, the target of rapamycin signaling pathway was found to be involved in starch accumulation *via* mediating phosphorylation of a glycogenin-like protein (CmGLG1) in the unicellular red alga *Cyanidioschyzon merolea*, and overexpression of *CmGLG1* resulted in 4.7-fold increase in the starch content (Pancha et al., 2019a; Pancha et al., 2019b). An earlier study showed knocking out a *Zn (II)_2_Cys_6_* gene improved partitioning of the total carbon to lipid from 20 to 40–55% in *Nannochloropsis* (Ajjawi et al., 2017). This study revealed an unprecedented regulator which is the homologous to the bacterial GNAT PA4794 identified from *P. aeuruginosa*. The *GNAT* gene family include a large family of enzymes that catalyze the transfer of an acyl moiety from acyl-CoA to a variety of substrates and involve in diverse biological processes (Salah Ud-Din et al., 2016). Though PA4794 was found to be capable of selectively acetylating the Nε group of the C-terminal lysine of peptides and small molecules such as chloramphenicol, the *in vivo* function of this protein has remained uncharacterized (Majorek et al., 2013). It is noteworthy that PA4794 is remotely related to the RimI ribosomal protein acetyltransferase. Thus, it is necessary to perform proteomics analysis on the OE lines constructed in this study to address whether the role of CrGNAT19 was involved in global translational regulation or responsible for acetylation of specific proteins.

In addition to altering carbon partitioning in *C. reinhardtii*, overexpression of CrGNAT19 led to substantial enhancement in biomass production under N-depleted conditions. Up to now, increasing algal biomass production has been largely pursued through enhancing photosynthesis efficiency, such as attenuating the light-harvesting antenna complexes (LHC) in particular. For the *C. reinhardtii* mutant NAB1* with the LHC size reduced by 10–17%, the growth rate during log phase and maximal dry weight under continuous high-light conditions was improved by 50 and 37%, respectively, as compared to the control (Beckmann et al., 2009). The LHC knock-down mutant Stm3LR3 also exhibited the fast-growing phenotype under high-light conditions (Mussgnug et al., 2007). However, the growth potential of these mutants under nutrient depletion conditions remain uncharacterized, and thus it is unclear whether they could be used to indirectly improve the production of starches or lipids, both of which are enormously accumulated under N-depletion conditions. Interestingly, similar to *CrGNAT19* OE lines, the *C. reinhardtii* mutant *std1* deficient in a plant-specific tyrosine-phosphorylation-regulated kinase was capable of accumulating higher levels of starch and achieving higher biomass productivity than wild-type under N-depletion conditions (Schulz-Raffelt et al., 2016). In contrast to these hyper-starch mutants including *CrGNAT19* and *std1*, the biomass production under N-depleted conditions was significantly suppressed in a number of starchless and low starch mutants of *C. reinhardtii* (Li et al., 2010b). These findings taken together indicated that starch biosynthesis can positively regulate the photosynthesis process under N-depletion in *C. reinhardtiii*. It is noteworthy that the function of CrGNAT 19 remained to be investigated under various trophic modes (i.e. photoautotrophic and heterotrophic) in order to fully demonstrate its biotechnical significance.

Taken together, this study uncovered the role of *CrGNAT19* in regulation of carbon partitioning in *C. reinhardtii*. Moreover, overexpression of this gene can drastically enhance the microalgal biomass and starch production, indicative of its great potential in genetic engineering of microalgae for production of starch and other bioproducts as well. We suspect the expression of CrGNAT19 could be up-regulated by the enhanced TAG biosynthesis mediated by multiple metabolic pathways (e.g. prokaryotic and eukaryotic pathways) thereby playing a central role in regulation of carbon partitioning. Identification of the substrate(s) and targeted pathway(s) for such an unprecedented regulator is underway.

## Data Availability Statement

The original contributions presented in the study are publicly available. This data can be found here: NCBI, accession number PRJNA587114.

## Author Contributions

ZL, LC, DH, and QH contributed conception and design of the study. ZL and LZ organized the database. ZL and LZ performed the statistical analysis. DH and ZL wrote the first draft of the manuscript. DH and ZL wrote sections of the manuscript. All authors contributed to the article and approved the submitted version. ZL and LC equally contributed to this work.

## Funding

This work was supported by The National Key R&D Program of China (2018YFA0902500) and Chinese Academy of Sciences (ZDRW-ZS-2017-2).

## Conflict of Interest

The authors declare that the research was conducted in the absence of any commercial or financial relationships that could be construed as a potential conflict of interest.
