# Supplementary figures and images for: Identification and Biotechnical Potential of a Gcn5-Related N-Acetyltransferase Gene in Enhancing Microalgal Biomass and Starch Production
Source: Front Plant Sci. 2020 Aug 28;11:544827. doi: 10.3389/fpls.2020.544827 (PMC7483765; doi:10.3389/fpls.2020.544827)

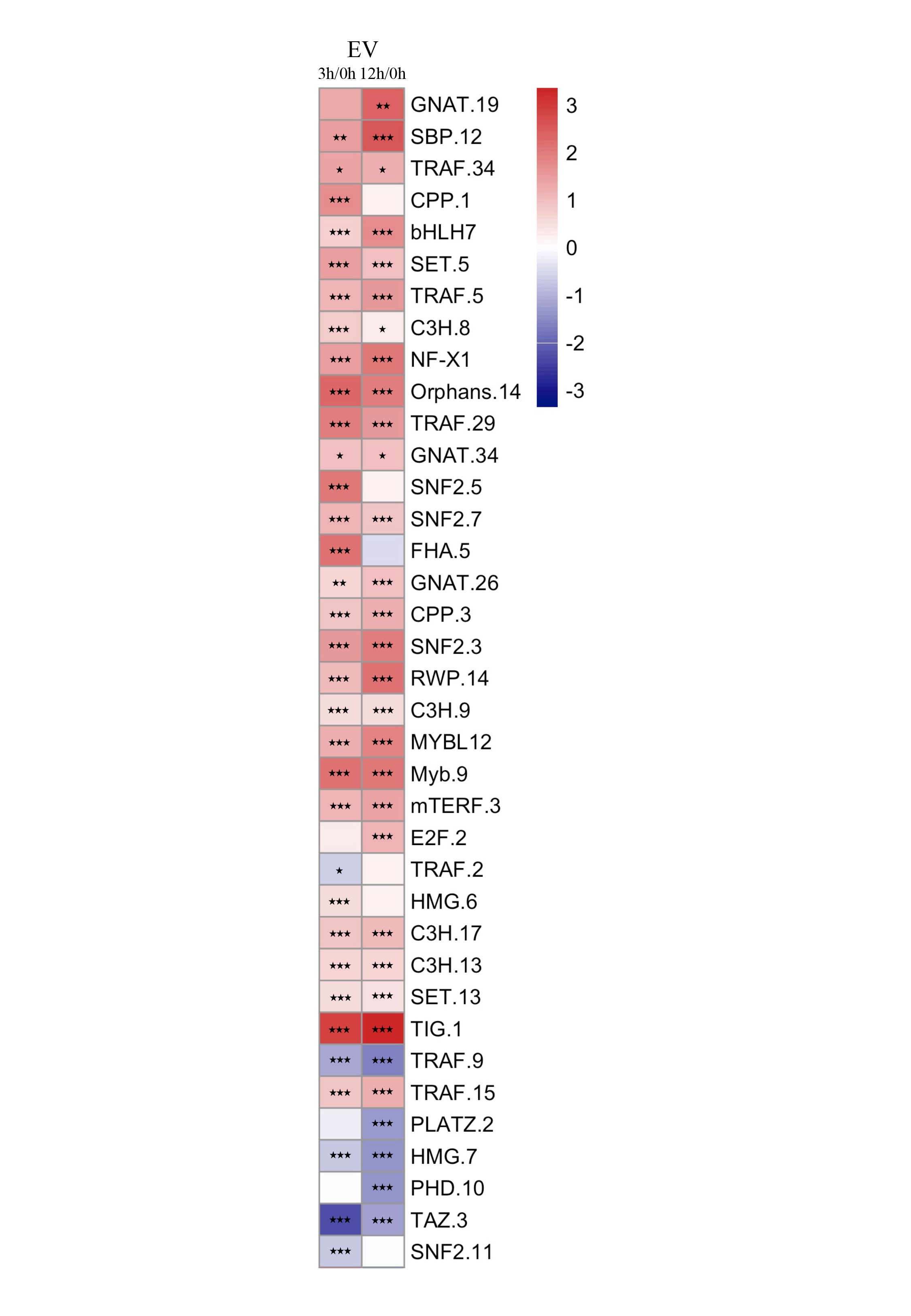

Supplement: Supplementary file 1 [file Image_1.jpeg]
