# Supplementary material for: Identification and Biotechnical Potential of a Gcn5-Related N-Acetyltransferase Gene in Enhancing Microalgal Biomass and Starch Production
Source: Front Plant Sci. 2020 Aug 28;11:544827. doi: 10.3389/fpls.2020.544827 (PMC7483765; doi:10.3389/fpls.2020.544827)
Supplement: Supplementary file 2 [file Table_1.docx]

**Supplemental Figure Legends**

Figure S1. Transcriptional dynamics of the differentially expressed transcription factor/regulator encoding genes in the P4 empty vector lines. Fold-changes over 12 h upon the onset of N-depletion stress were calculated as log_2_ (3h/0h) and log_2_ (12h/0h) (EV, P4 empty vector lines) and displayed in the heat map. Statistical significances of the differentially expressed genes were determined by a false discovery rate (FDR)-corrected *p* value (*, *p* < 0.05; **, *p* < 0.01; ***, *p* < 0.001).
